# P351: Undesirable adverse events of the thoracic surgery department of the institute of cardiology of Abidjan

**DOI:** 10.1186/2047-2994-2-S1-P351

**Published:** 2013-06-20

**Authors:** M Adeoti, O Oyourou

**Affiliations:** 1 University Felix Houphouet Boigny, Côte d'Ivoire; 2 National Institute of Public Health, Abidjan, Côte d'Ivoire

## Introduction

In the health care system, at least 10% of the admissions at the hospital involve adverse events (AE) for inpatients, half of them being regarded as avoidable. These events, which can have serious consequences (death, handicap), have a considerable economic impact.

## Objectives

To analyze adverse events occurring in the thoracic surgery department of the ICA and to describe the causes for each type of adverse event.

## Methods

This is a retrospective study of serious adverse events occurring in the thoracic surgery department of the Institute of Cardiology of Abidjan. The study involved the analysis of patient records from 2006-2007[ha1] . An interview was also conducted with the head of service.

## Results

Of the 113 hospitalized patients, only 80 patients had complete and available records. Thirty-four adverse events were identified and analyzed following an interview with the heads of thoracic surgery.32 AE occurred during the hospitalization; two of these were unavoidable. 73% of AE were serious and the incidence rate of AE in the thoracic surgery department is of 6.89%. 19 patients suffered an AE.[ha1] The AE average per patient during care in thoracic surgery is 1.5. Serious adverse events related to invasive acts are the types most observed (38.24%) in thoracic surgery. 52.9% of AE were preventable and 41.8% likely preventable. 68% of identified AE were related to the technical aspects of the care of patients that is to say the task.

## Conclusion

This study showed the existence of several failures related to the organization, the task, the environment, the institution and the team.

The challenge for the ICA is, within the limits of current knowledge and techniques, to ensure maximum safety for patients regarding their clinical risk.

## Disclosure of interest

None declared

